# Impact of the COVID-19 Pandemic on the Severity and Early Postoperative Outcomes of Acute Appendicitis

**DOI:** 10.7759/cureus.42923

**Published:** 2023-08-03

**Authors:** Hai V Nguyen, Loc H Tran, Tuan H Ly, Quang T Pham, Vu Q Pham, Ha N Tran, Loc T Trinh, Thien T Dinh, Dinh T Pham, Tuong Anh Mai Phan

**Affiliations:** 1 Department of General Surgery, University of Medicine and Pharmacy at Ho Chi Minh City, Ho Chi Minh City, VNM; 2 Department of Digestive Surgery, Nhan Dan Gia Dinh Hospital, Ho Chi Minh City, VNM; 3 Department of General Surgery, University of Medicine and Pharmacy at Ho Chi Minh City, Ho Chi Minh, VNM

**Keywords:** covid-19 pandemic, laparoscopic appendectomy, open appendectomy, non-operative management, acute appendicitis

## Abstract

Background

The coronavirus disease 2019 (COVID-19) pandemic caused changes in surgical practice. For acute appendicitis (AA), measures to control the pandemic might hinder patients from seeking medical care timely, resulting in increasing severity, postoperative complications, and mortality. This study aimed to investigate whether the COVID-19 pandemic had a negative impact on the severity and postoperative outcomes of patients with AA.

Methodology

We retrospectively reviewed medical records of AA patients treated operatively at Nhan Dan Gia Dinh Hospital hospital from June 1st to September 30th in three consecutive years: pre-pandemic (2019)/Group 1, minor waves (2020)/Group 2, and major wave (2021)/Group 3 (2021). Data were collected focusing on the duration of symptoms, severity of AA, time from admission to operation, postoperative complications, and mortality.

Results

There were 1,055 patients, including 452 patients in Group 1, 409 in Group 2, and 194 in Group 3. The overall number of patients decreased mainly in non-complicated AA. The percentages of hospital admission after 24 hours gradually increased (20.8%, 27.9%, and 43.8%, p < 0.05). The percentages of complicated AA in Group 2 and Group 3 were statistically higher than in Group 1 (39% and 55% vs. 31%, p < 0.05). Waiting time for operation increased to five hours during the major wave. Laparoscopic appendectomy was performed in 98-99% of AA patients during the pandemic, with an early postoperative complication rate of 5-9% and a mortality rate of 0.2-1%.

Conclusions

Although the percentages of hospital admission after 24 hours and complicated AA increased, laparoscopic appendectomy was still feasible and effective and should be maintained as the standard management for AA during the COVID-19 pandemic.

## Introduction

Coronavirus disease 2019 (COVID-19) is the name of an infectious condition caused by severe acute respiratory syndrome coronavirus 2 (SARS-CoV-2) which was first identified in the beginning of 2020 in China. After being declared by the World Health Organization (WHO) as a pandemic on March, 11th 2020, the COVID-19 pandemic rapidly spread and profoundly influenced healthcare systems worldwide [1,2]. To control the pandemic, many measures were implemented such as staying at home, social distancing, and lockdown. Although these measures may help in restricting the spread of the COVID-19 pandemic, they may also hinder the usual access of patients to healthcare, even in emergency situations. In addition, patients in the COVID-19 era might present to the hospital late due to their anxiety and fear of being infected with COVID-19 [3]. During the COVID-19 pandemic waves, some guidelines encouraged the postponement of elective surgeries and transferral to non-operative management for surgical emergencies which were in uncomplicated stages such as acute appendicitis (AA) and colonic diverticulitis [1,4].

AA is the most common cause of abdominal emergencies with a lifetime risk of 7-8% [2,4,5]. Although recent literature has accepted non-operative management as an alternative for uncomplicated AA, especially in the time of the COVID-19 pandemic, the standard management for AA is an appendectomy (laparoscopic is currently preferred to an open approach) [4,5]. Theoretically, late admission from the onset of symptoms may increase the severity of AA, and then surgery at a complicated stage may also increase postoperative complications and death. Practically, studies around the world about the impact of the COVID-19 pandemic on severity and early outcomes after surgery for AA have reported different results. Some recognized a decrease in the total number of AA cases, an increase in the number of complicated AA cases, and an increase in the rate of postoperative complications [6-8]. Others showed there were no differences in terms of the length of preoperative symptoms, the distribution of complicated versus uncomplicated appendicitis, and the rate of postoperative complications [9,10]. These differences were not adequately explained and may relate to the variability of the COVID-19 pandemic situation in different countries.

Our hypothesis was that the COVID-19 pandemic, with a possible delay in access to emergency service, may impact the severity and early postoperative outcomes of AA. This study aimed to identify the time from onset of symptoms to admission and operation, the rate of complicated AA, early postoperative complications, and mortality of patients with AA in the pre-COVID-19 period during the minor waves of the COVID-19 pandemic and the major wave of the COVID-19 pandemic.

## Materials and methods

Study design

This retrospective study was performed at Nhan Dan Gia Dinh Hospital and was approved by both Institutional Review Boards of the University of Medicine and Pharmacy at Ho Chi Minh City and Nhan Dan Gia Dinh Hospital.

**Figure 1:**
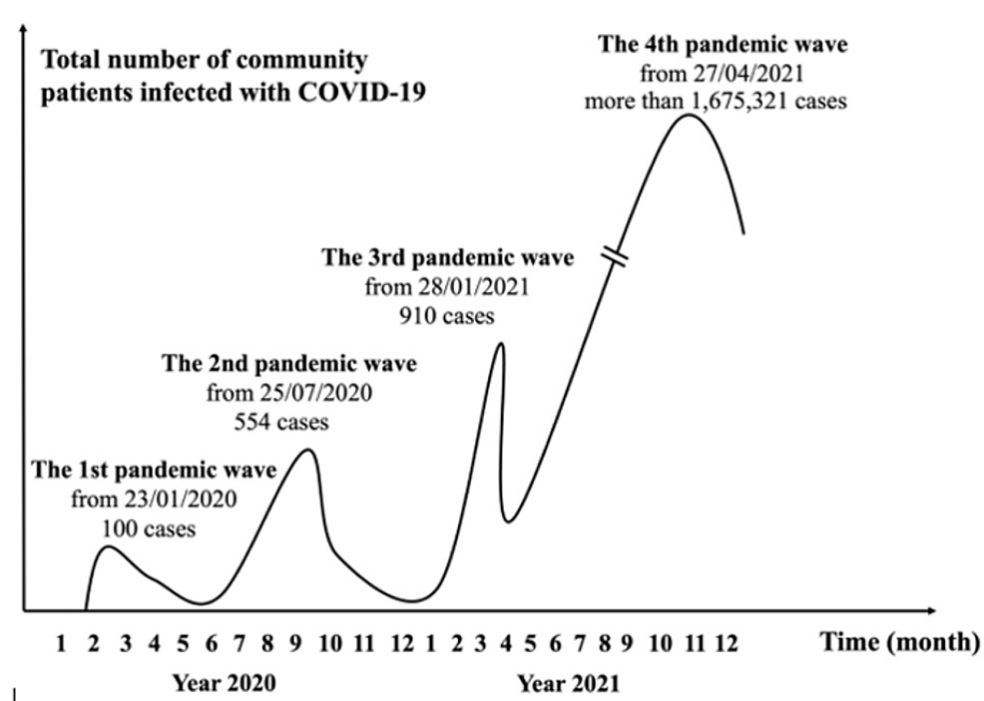
The waves of the COVID-19 pandemic and the number of cases in Vietnam. Data from Minh et al. [11] and updated up to December 2021.

The first case of COVID-19 in Vietnam was detected on January 23, 2020. In 2020 and the first quarter of 2021, there were three minor waves of the COVID-19 pandemic (with 100, 554, and 910 cases) (Figure 1). From April 27, 2021, the fourth wave (the major wave or peak) of the COVID-19 pandemic occurred across Vietnam, mainly in the South, with more than 1.6 million cases [11]. With a rapid increase in the number of infected cases despite social distancing and intensive vaccination, a lockdown was applied strictly from June 1 to September 30, 2021.

**Figure 2:**
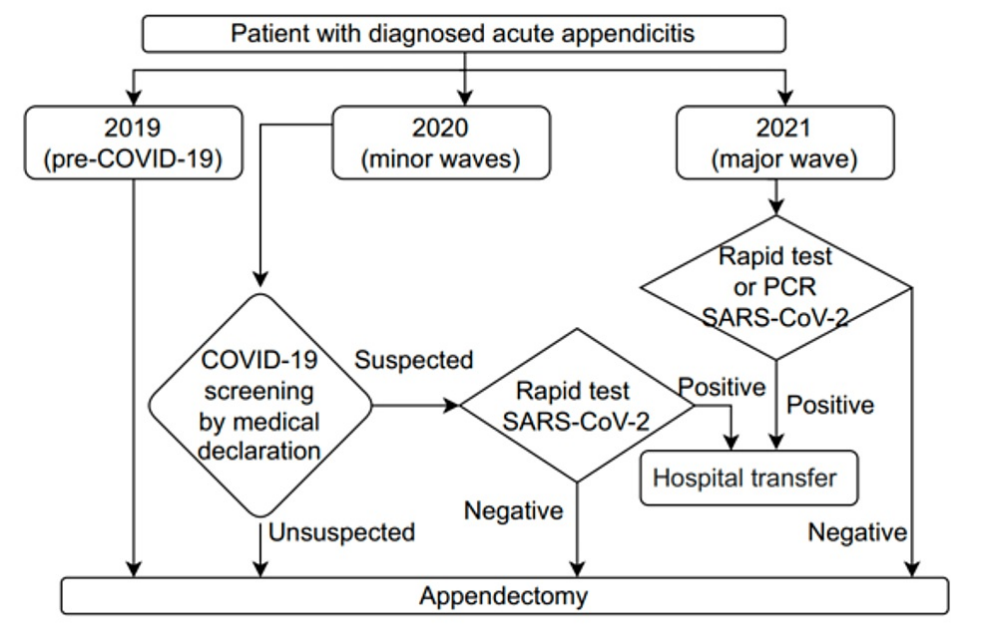
Preoperative screening for COVID-19 in patients with diagnosed acute appendicitis.

Our hospital is a tertiary general hospital and was among the hospitals in Ho Chi Minh City (HCMC) that had partially changed to serve COVID-19 patients. In 2020, every patient had to perform a medical declaration, but a rapid test for COVID-19 was indicated only for suspected cases. In 2021, we performed rapid tests and reverse transcription polymerase chain reaction (RT-PCR) tests for COVID-19 more often for patients. Only surgical patients (including AA patients) with negative results for COVID-19 were treated at our hospital. Surgical patients with positive results for COVID-19 were transferred to hospitals designed to treat COVID-19 patients (Figure 2).

Study population

Patients who were treated for AA in the same period (from June 1 to September 30) of three consecutive years were chosen for the study. Group 1 included AA patients in the pre-pandemic period (2019), Group 2 included AA patients at the same time in 2020, and Group 3 included AA patients at the same time in 2021. Diagnosis of AA was based on clinical characteristics, laboratory tests, and imaging investigation (ultrasound and/or CT), and was confirmed by postoperative pathologic results. We did not choose AA patients who were treated by non-operative management because of the uncertainty of diagnosis. In addition, this strategy is not accepted widely in our country. We also excluded pediatric patients because we do not have a pediatric surgery department.

Data collection and variable definitions

Data collected from the medical record of each patient included age, gender, duration from symptom onset to admission and operation, waiting time for COVID-19 tests, grade of AA, surgical approach, early postoperative complications, mortality, hospital stay after operation, re-admission, and re-operation.

Appendicitis severity was classified according to the American Association for the Surgery of Trauma (AAST) grading score (2016), as described by surgeons [12]. Uncomplicated appendicitis was defined as grade 1 (acutely inflamed appendix, intact), whereas complicated appendicitis was defined as grade 2-5 (gangrenous or perforated appendix).

Early postoperative complications were defined as complications that occurred 30 days after the operation or in the same admission (if the hospital stay was over 30 days), including wound infection, intra-abdominal abscess, bowel obstruction, and others. Re-admission and re-operation data were collected 30 days after the operation from the information stored in computerized patient archives.

Statistical analysis

Data were analyzed using SPSS for Windows version 20 (IBM Corp., Armonk, NY, USA). Continuous normally distributed variables were expressed as means and standard deviations (SDs). Continuous non-normally distributed variables were expressed as median and interquartile ranges. Categorical variables were expressed as frequencies and percentages. Continuous data were tested for normal distribution and compared using the independent t-test or the Mann-Whitney U test, as appropriate. Categorical variables were compared using the chi-square test and Fisher’s exact test, as appropriate. All p-values were based on two-sided tests, and p-values <0.05 were considered statistically significant.

## Results

A total of 1,055 patients were enrolled in this study, including 452 patients in Group 1 (2019), 409 patients in Group 2 (2020), and 194 patients in Group 3 (2021). Demographic data (age, gender) showed no difference between the three groups. The mean age was about 40 years old, and the male:female ratio was about 1:1 (Table 1). There was a 9.5% decrease in the total number of AA patients in Group 2 in comparison to Group 1, and a 57% decrease in the total number of AA patients in Group 3 in comparison to Group 1. Analyzing data according to grades of AA as in Table 1, we found that the decrease in Group 3 (the major wave of the pandemic) was mainly driven by a dramatic decrease in uncomplicated AA patients. Meanwhile, the absolute number of complicated AA patients in the three periods showed slight changes (141, 159, and 107 cases).

**Table 1:** Patient characteristics, duration, and severity of acute appendicitis. Data expressed as mean ± SD or median (first quartile; third quartile) for continuous variables and n (%) for nominal variables. AAST: American Association for the Surgery of Trauma; C: chi-square test; F: Fisher’s exact test; T: t-independent test; M: Mann-Whitney U test

	Group 1 (N1 = 452)	Group 2 (N2 = 409)	P-value G1-G2	Group 3 (N3 = 194)	P-value G1-G3
Age (years)	39.8 ± 16.9	41.2 ± 17.1	0.229^T^	40.1 ± 15.0	0.837^T^
Sex, n (%)			0.590^C^		0.278^C^
Male	205 (45.4)	193 (47.2)		97 (50)	
Female	247 (54.6)	216 (52.8)		97 (50)	
Duration of symptoms (hours)					
≤24 hours, n (%)	358 (79.2)	295 (72.1)	0.015^C^	109 (56.2)	0.001^C^
>24 hours, n (%)	94 (20.8)	114 (27.9)		85 (43.8)	
Duration from hospital admission to operation (hours)	8 [6; 11]	8 [6; 12]	p = 0.140^M^	13 [10.8; 16]	<0.001^M^
Duration of waiting for COVID-19 laboratory test result (hours)	-	-		4 [0; 7]	
Operative AAST description of appendicitis (2016), n(%)			0.040^C^		<0.001^C^
Grade 1	311 (68.8)	250 (61.2)		87 (44.8)	
Grade 2	19 (4.2)	16 (3.9)		6 (3.1)	
Grade 3	72 (15.9)	78 (19.1)		62 (32)	
Grade 4	20 (4.4)	16 (3.9)		19 (9.8)	
Grade 5	30 (6.6)	49 (11.9)		20 (10.3)	

Regarding the duration of symptoms (from onset to hospital admission), the percentage of patients admitted after 24 hours in Group 2 was significantly high in comparison to Group 1 (27.9% vs 20.8%, chi-square test, p = 0.015). Similarly, the percentage of patients admitted after 24 hours in Group 3 was significantly high in comparison to Group 1 (43.8% vs 20.8%, chi-square test, p < 0.001). The median time from admission to the operation of patients in Group 1 and Group 2 was eight hours, and there was no difference between the two groups. However, the median time from admission to operation in Group 3 was longer than that in Group 1 and Group 2 by approximately five hours (13 hours vs. 8 hours, Mann-Whitney U test, p < 0.001). Waiting time for operation in Group 3 patients increased by approximately five hours.

**Figure 3:**
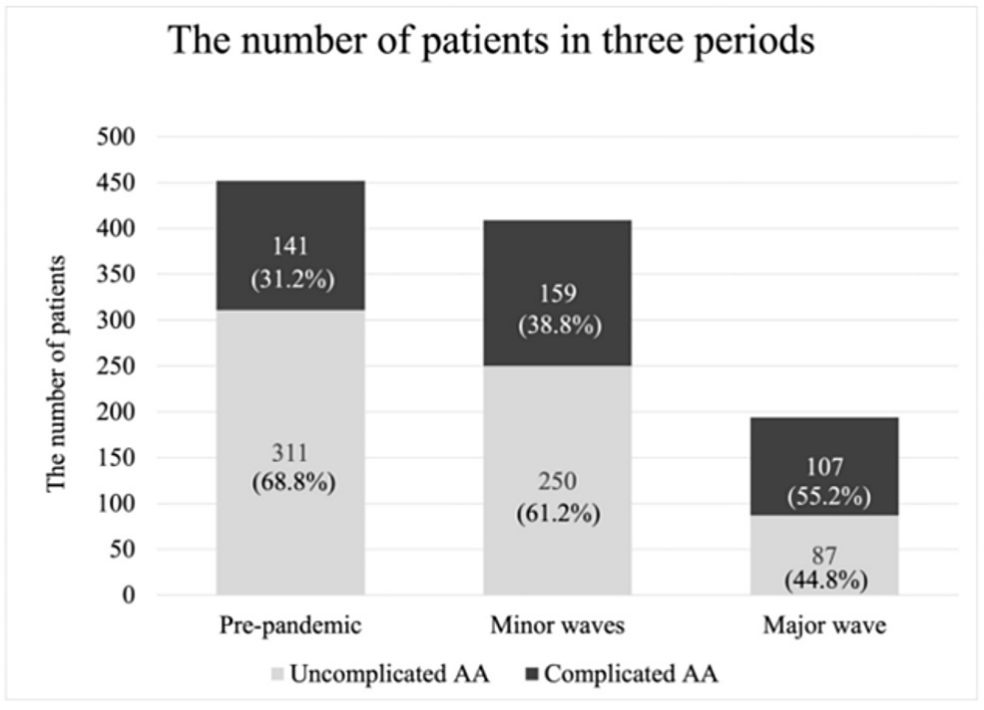
Distribution of uncomplicated and complicated AA patients across the three periods of the COVID-19 pandemic. AA: acute appendicitis

Regarding the severity of AA, results in Table 1 and Figure 3 show that the percentage of complicated AA in Group 2 was statistically higher than the percentage of complicated AA in Group 1 (39% vs 31%, chi-square test, p = 0.040). Similarly, the percentage of complicated AA in Group 3 was statistically higher than the percentage of complicated AA in Group 1 (55% vs 31%, chi-square test, p < 0.001).

As mentioned above, all our AA patients in the three groups were treated by appendectomy after they had a negative result for COVID-19 screening tests. Patients with positive COVID-19 screening tests were transferred to the hospital designed for treating COVID-19 patients. With that strategy, laparoscopic appendectomy was performed as usual for almost all AA patients in our hospital (98-99%), even in the major wave of the COVID-19 pandemic (Group 3) (Table 2). The rate of open appendectomy was only 0.7-2.1%.

**Table 2:** Early postoperative outcomes of patients with acute appendicitis. Data expressed as median [first quartile; third quartile] for continuous variables and n (%) for nominal variables. C: chi-square test; F: Fisher’s exact test; T: t-independent test; M: Mann-Whitney U test; *: Death.

	Group 1 (N1 = 452)	Group 2 (N2 = 409)	P-value G1-G2	Group 3 (N3 = 194)	P-value G1-G3
Surgical access, n (%)			0.176^C^		0.759^F^
Laparoscopic	444 (98.2)	406 (99.3)		190 (97.9)	
Open or conversion	8 (1.8)	3 (0.7)		4 (2.1)	
Complications, n (%)			0.417^C^		0,274^C^
No complication	418 (92.5)	372 (91)		184 (94.9)	
Multiorgan failure	0 (0)	1 (0.2) *		2 (1) *	
Wound infection	25 (5.5)	18 (4.4)		2 (1)	
Intra-abdominal abscess	9 (2)	16 (3.9)		5 (2.6)	
Bowel obstruction	0 (0)	2 (0.5)		1 (0.5)	
Total complications	34 (7.5)	37 (9)		10 (5.1)	
Mortality	0 (0)	1 (0.2)	0.475^F^	2 (1)	0.090^F^
Hospital stay after operation (days)	2 [1;3]	2 [1;3]	0.125^M^	2 [1;3]	0.055^M^
Re-admission, n (%)			0.431^F^		1.000^F^
No	450 (99.6)	405 (99)		194 (100)	
Yes	2 (0.4)	4 (1)		0 (0)	
Re-operation, n (%)					
No	452 (100)	409 (100)		194 (100)	
Yes	0	0		0	

The postoperative course of AA patients in Group 1 and Group 2 was similar in terms of early complications. The total rate of complications was 7.5% for Group 1 and 9% for Group 2 (chi-square test, p = 0.417). There was a decrease in the total rate of early complications in Group 3 (5.1%); however, in comparison to Group 1, this decrease was not significant (chi-square test, p = 0.274). The rates of postoperative intra-abdominal abscess in the three groups were also not significantly different (range = 2.0-3.9%).

We had three deaths, including one patient in Group 2 (0.5%) and two patients in Group 3 (1%). All were elderly patients (64, 68, and 70 years old) with severe comorbidities, and were diagnosed as having diffuse peritonitis with preoperative septic shock. After intensive resuscitation, open appendectomy and peritoneal cleansing were performed. They died on postoperative day (POD) seven, 15, and 23 because of unrecoverable septic shock and multiorgan failure. Rapid and RT-PCR tests excluded that they were concomitantly infected by SARS-CoV-2.

Regarding hospital stay after the operation, the median time in the three groups was two days, and there was no significant difference (Mann-Whitney U test, p = 0.125, and p = 0.055). There were two patients in Group 1 (0.4%) and one patient in Group 2 (1%) re-admitted with early complications, but they responded well to medical treatment and no re-operation was necessary.

## Discussion

This study was performed in the most seriously COVID-19-affected region in Vietnam. By choosing the same period in three consecutive years corresponding to the stages of the COVID-19 pandemic (pre-pandemic, minor waves, and major wave) to investigate the impact, we thought that we could avoid seasonal variations in the incidence of AA in our analysis. Demographic data (mean age, gender) of AA patients in our study were similar to the results from other studies [2,5,8,13].

Many authors reported a decrease, which varied from 5.5% to 46%, in the total number of AA patients during the COVID-19 pandemic [7,8,10,14-17]. This change was mainly attributable to the drop in the number of non-complicated AA patients, while the number of complicated AA patients remained stable [14]. Our study confirmed this change. The decrease in the total number of AA patients during the major wave of the COVID-19 pandemic in our study (Group 3) was higher than that reported in other studies (57%). We think that strict lockdown along with the patient’s fear of infecting SARS-CoV-2 when visiting the hospital might explain this decrease. Moreover, the re-allocation of our hospital for not performing surgery in patients with positive COVID-19 screening tests might be a contributing factor. In another meaning, a significant decrease in the number of noncomplicated AA patients without a corresponding increase in the absolute number of complicated AA patients during the pandemic suggested that noncomplicated AA might be a mild entity, and surgical treatment could not be mandatory for all cases. Some cases of early noncomplicated AA could have spontaneous resolution or respond well with non-operative treatment [7,16,18].

Regarding the duration of symptoms, our data showed the percentage of patients admitted hospital more than 24 hours after onset was statistically higher during the pandemic (both in the minor waves and in the major wave periods) in comparison to the pre-pandemic period. The data from Scheijmans et al. showed an increase in the percentage of patients who had a duration of symptoms more than 24 hours from 56.2% to 61.1% (p = 0.048), especially in elderly patients and complicated AA patients [8]. An et al. recognized the median duration of symptoms before admission was six hours longer during the pandemic compared to the pre-pandemic period (18 vs. 24 hours) [6]. Meanwhile, Tankel et al. and Francombe et al. did not find any significant difference in the duration of symptoms (1.5 vs. 1.8 days, and 2.2 vs 2.3 days, respectively) [10,19]. Various durations of symptoms in published reports worldwide might relate to many factors, such as the severity of the regional COVID-19 pandemic and strictness of control policies, patient consciousness, and the healthcare system.

When grading the severity of AA according to the AAST definition, our percentage of complicated AA (grade 2-5) both in the minor waves and the major wave of the COVID-19 pandemic were statistically higher (8% and 24%) than in the pre-pandemic period. Although some authors did not report this increase [10,16,20], data from many other studies are similar to our findings [2,6,8,19,21]. The increase in the percentage of complicated AA might have been attributed to delayed hospital admission in the pandemic context.

During the COVID-19 pandemic, there were some changes in AA treatment. Data from studies showed an increase in conservative management and a decrease in appendectomy [4,5]. The World Society of Emergency Surgery guidelines recommended initial non-operative management for non-complicated AA, especially in regions with resource constraints due to the COVID-19 pandemic. In addition, for patients with complicated AA who needed operation (abscesses failed with non-operative management and percutaneous drainage, or peritonitis), open appendectomy was recommended instead of laparoscopic appendectomy to minimize intraoperative virus dissemination [1,22].

Until now, non-operative management for non-complicated AA has not been widely accepted in our country, hence, we treated almost AA patients by appendectomy as soon as possible, even during the COVID-19 pandemic. Because our hospital was reallocated to operate only on AA patients who had negative COVID-19 screening tests, we chose the laparoscopic approach as usual. We did not utilize any special device for gas filtration as recommended. We observed the high rates of successful laparoscopic appendectomy for treating AA patients in both pandemic periods (99% in Group 2, and 98% in Group 3), and these rates were not different in comparison to the pre-pandemic period. The rate of open appendectomy (including conversion to open surgery) was stable at 1-2% across periods. Apart from data from El Nakeeb et al. with an increased rate of open appendectomy from 56.5% (pre-COVID-19 period) to 58.7% (COVID-19 period) [15], the results from other studies aligned with our result regarding the high rate of successful laparoscopic appendectomy during the COVID-19 pandemic (70-99%) [2,5,6,8,9,17]. We think that laparoscopic appendectomy had been feasible and safe for almost AA patients with negative COVID-19 screening tests during the pandemic.

Regarding median time from hospital admission to operation, we noticed an additional approximately five hours in the major wave, but no change in the minor waves of the COVID-19 pandemic. This prolongation was necessary for screening SARS-CoV-2 infection which was mandatory for every surgical patient in our hospital at the peak of the pandemic. Kim et al. also reported an increase of eight hours in the median time from admission to operation during the COVID-19 pandemic in comparison to the pre-pandemic period [9]; however, some other authors did not find any change in this time (five to nine hours) [2,6,20]. Variations in this time might relate to the strategy of performing safe surgical care during the COVID-19 pandemic in each country.

There were some variations in the rate of early complications after appendectomy during the COVID-19 pandemic among studies worldwide. While El Nakeeb et al., with open appendectomy, accounted for nearly 60% of cases, reported an increase in the complication rate in comparison to the pre-pandemic period (11.3% vs. 5.8%, p = 0.0001) [15], other authors, with laparoscopic appendectomy accounting for the majority of cases, reported the unchanged complication rate (4.6-20.6%) [6,8,9,19,20]. Mortality was also unchanged (0-0.3%) [2,17-20]. Although the percentage of complicated AA in our study was higher in both COVID-19 pandemic periods, we did not find any significant increase in the rate of early postoperative complications and mortality (5-9% and 0-1%, respectively) across periods. Our incidences of postoperative intra-abdominal abscesses during the COVID-19 pandemic (minor waves and major waves) did not increase in comparison to the pre-pandemic period and were similar to the incidence of postoperative intra-abdominal abscesses from the study by Mulita et al. (3.7%) [23]. All deaths in our study occurred in patients with septic shock, diffuse peritonitis, and severe comorbidities.

Our data also aligned with data from other studies about the rate of re-admission (range = 0-7.7%) and the rate of re-operation (range = 0-2.5%) [6,10,14,15,19]. Although some authors recognized a statistical increase in these rates in comparison to the pre-pandemic period [6,15], many authors did not find any significant difference [9,14,19]. In fact, the majority of early postoperative complications in our study and other studies were wound infections and intra-abdominal abscesses. Some patients with mild or moderate complications might treat themselves or with consultancy and restricted visiting the hospital during the peak of the COVID-19 pandemic.

Until now, there are no studies regarding the surgical management of COVID-19-associated AA with sample sizes large enough, perhaps because the incidence of COVID-19 infection in AA patients is quite low at approximately 1.8% [14]. Meanwhile, concerns regarding the safety of appendectomy in AA patients who are concomitantly infected with SARS-CoV-2 need more evidence to guide safe and effective surgical practice during the pandemic. Recently, Forssten et al., in a prospective multi-institutional study about appendectomy for AA in 4,047 patients (including 70 active and 116 prior SARS-CoV-2 infection patients), did not find any association between SARS-CoV-2 infection and postoperative complications as well as the length of hospital stay [24]. This finding, together with the results from our study and the above-mentioned studies, suggests that we can maintain laparoscopic appendectomy as the standard management for AA during the COVID-19 pandemic.

Although we selected only AA patients who were treated by operative management and were confirmed by histologic results to avoid uncertain diagnoses, our study had some limitations. First, data were collected retrospectively and depended on medical records, resulting in bias. Second, during the peak of the pandemic, it was impossible to track all patients after discharge as usual. Therefore, some patients with postoperative complications, or even postoperative COVID-19 infection, who were not re-examined in the hospital might be missed. Third, we did not treat AA patients who had concomitant COVID-19 infection and did not include AA patients who were treated non-operatively, so our results did not comprehensively reflect the management of AA during the COVID-19 pandemic.

## Conclusions

During the COVID-19 pandemic, there was a significant decrease in the overall number of AA patients. This decrease was mainly in the number of non-complicated AA patients; hence, the percentage of complicated AA increased. Social distancing and lockdown resulted in increasing the rate of patients admitted to the hospital more than 24 hours after onset. Moreover, COVID-19 screening tests prolonged the waiting time for the operation. Even so, a laparoscopic appendectomy could still be feasible and effective for almost all AA patients, with an unchanged rate of early postoperative complications and mortality. Whenever and wherever possible, laparoscopic appendectomy should be maintained as the standard management for AA.

## References

[1] De Simone B, Chouillard E, Sartelli M (2021). The management of surgical patients in the emergency setting during COVID-19 pandemic: the WSES position paper. World J Emerg Surg.

[2] Lescinska AM, Sondore E, Ptasnuka M, Mukans M, Plaudis H (2023). The course and surgical treatment of acute appendicitis during the first and second wave of the COVID-19 pandemic: a retrospective analysis in university affiliated hospital in Latvia. Medicina (Kaunas).

[3] Mulita F, Vailas M, Tchabashvili L, Liolis E, Iliopoulos F, Drakos N, Maroulis I (2021). The impact of the COVID-19 outbreak on emergency surgery: a Greek emergency department experience. Prz Gastroenterol.

[4] Köhler F, Müller S, Hendricks A (2021). Changes in appendicitis treatment during the COVID-19 pandemic - a systematic review and meta-analysis. Int J Surg.

[5] Somers K, Abd Elwahab S, Raza MZ (2021). Impact of the COVID-19 pandemic on management and outcomes in acute appendicitis: should these new practices be the norm?. Surgeon.

[6] An S, Kim HR, Jang S, Kim K (2022). The impact of the coronavirus disease - 19 pandemic on the clinical characteristics and treatment of adult patients with acute appendicitis. Front Surg.

[7] Köhler F, Acar L, van den Berg A (2021). Impact of the COVID-19 pandemic on appendicitis treatment in Germany-a population-based analysis. Langenbecks Arch Surg.

[8] Scheijmans JC, Borgstein AB, Puylaert CA (2021). Impact of the COVID-19 pandemic on incidence and severity of acute appendicitis: a comparison between 2019 and 2020. BMC Emerg Med.

[9] Kim CW, Lee SH (2021). Impact of COVID-19 on the care of acute appendicitis: a single-center experience in Korea. Ann Surg Treat Res.

[10] Tankel J, Keinan A, Blich O (2020). The decreasing incidence of acute appendicitis during COVID-19: a retrospective multi-centre study. World J Surg.

[11] Minh LH, Khoi Quan N, Le TN, Khanh PN, Huy NT (2021). COVID-19 timeline of Vietnam: important milestones through four waves of the pandemic and lesson learned. Front Public Health.

[12] Tominaga GT, Staudenmayer KL, Shafi S (2016). The American Association for the Surgery of Trauma grading scale for 16 emergency general surgery conditions: disease-specific criteria characterizing anatomic severity grading. J Trauma Acute Care Surg.

[13] Chaves CE, Girón F, Núñez-Rocha RE (2023). Variations in clinical course and surgical outcomes of acute appendicitis during COVID-19 Pandemic: a multicenter cohort study. BMC Surg.

[14] Ceresoli M, Coccolini F, Magnone S (2021). The decrease of non-complicated acute appendicitis and the negative appendectomy rate during pandemic. Eur J Trauma Emerg Surg.

[15] El Nakeeb A, Emile SH, AbdelMawla A (2022). Presentation and outcomes of acute appendicitis during COVID-19 pandemic: lessons learned from the Middle East-a multicentre prospective cohort study. Int J Colorectal Dis.

[16] Neufeld MY, Bauerle W, Eriksson E (2021). Where did the patients go? Changes in acute appendicitis presentation and severity of illness during the coronavirus disease 2019 pandemic: a retrospective cohort study. Surgery.

[17] Sartori A, Podda M, Botteri E, Passera R, Agresta F, Arezzo A (2021). Appendectomy during the COVID-19 pandemic in Italy: a multicenter ambispective cohort study by the Italian Society of Endoscopic Surgery and new technologies (the CRAC study). Updates Surg.

[18] Rosenthal MG, Fakhry SM, Morse JL (2021). Where did all the appendicitis go? Impact of the COVID-19 pandemic on volume, management, and outcomes of acute appendicitis in a nationwide, multicenter analysis. Ann Surg Open.

[19] Frankcombe D, Gauri N, Satchithanandha V (2022). Management of acute appendicitis during the COVID-19 pandemic: a retrospective cohort study. BMC Surg.

[20] Tsou HJ, Huang SS, Tsai CH (2022). Outcomes of laparoscopic appendectomy during the level 3 alert of the coronavirus disease 2019 pandemic in Taiwan: experience in a referral center. Formosan J Surg.

[21] Santone E, Izzo F, Lo K, Pérez Coulter AM, Jabbour N, Orthopoulos G (2022). Long-term results on the severity of acute appendicitis during COVID-19 pandemic. Surg Open Sci.

[22] Di Saverio S, Khan M, Pata F, Ietto G, De Simone B, Zani E, Carcano G (2020). Laparoscopy at all costs? Not now during COVID-19 outbreak and not for acute care surgery and emergency colorectal surgery: a practical algorithm from a hub tertiary teaching hospital in Northern Lombardy, Italy. J Trauma Acute Care Surg.

[23] Mulita F, Plachouri KM, Liolis E, Kehagias D, Kehagias I (2021). Comparison of intra-abdominal abscess formation after laparoscopic and open appendectomy for complicated and uncomplicated appendicitis: a retrospective study. Wideochir Inne Tech Maloinwazyjne.

[24] Forssten MP, Kaplan LJ, Tolonen M (2023). Surgical management of acute appendicitis during the European COVID-19 second wave: safe and effective. Eur J Trauma Emerg Surg.

